# Development and temporal validation of a calibrated machine learning model for in-hospital blood culture positivity using routine clinical data

**DOI:** 10.3389/fmed.2026.1956921

**Published:** 2026-10-09

**Authors:** Tao Huang, Zhenzhen Tan, Longyang Jiang, Xuping Yang, Shurong Wang, Yilan Huang

**Affiliations:** 1 Department of Pharmacy, The Affiliated Hospital, Southwest Medical University, Luzhou, China; 2 School of Pharmacy, Southwest Medical University, Luzhou, China; 3 Department of Pharmacy, The Hejiang People’s Hospital, Luzhou, China

**Keywords:** blood culture positivity, clinical risk stratification, electronic health records, machine learning, probability calibration, temporal validation, XGBoost

## Abstract

**Background:**

Among hospitalized patients who have already undergone blood culture collection, routinely available clinical information may support early risk stratification while microbiological results are pending. We developed and temporally validated a calibrated machine learning model for in-hospital blood culture positivity.

**Methods:**

This single-center retrospective study included 44,389 blood culture-related hospitalization records from January 2022 to September 2024. Records from 2022 to 2023 were used for model development and records from 2024 for temporal validation. The primary outcome was at least one laboratory-recorded positive blood culture between index collection and hospital discharge. A Platt-calibrated XGBoost model using demographic characteristics, routine laboratory variables, and missingness indicators was compared with procalcitonin-only and regression-based models. Performance was assessed using discrimination, calibration, threshold-specific metrics, decision curve analysis, and SHAP.

**Results:**

The cohort included 3,555 outcome-positive records (8.01%). In temporal validation, the final XGBoost model achieved a ROC-AUC of 0.798 (95% CI, 0.784–0.813), a PR-AUC of 0.266, a Brier score of 0.0619, and a calibration slope of 1.061. At the 5% threshold, sensitivity was 0.877, specificity 0.570, and negative predictive value 0.983. XGBoost showed a modest improvement over logistic regression with missingness indicators, while an exploratory *post hoc* Age + PCT + BMI logistic comparator achieved a ROC-AUC of 0.757. Performance was stable in the new-patient validation subset but decreased within adult and pediatric subgroups (ROC-AUC 0.679 and 0.663, respectively). Age, procalcitonin, and body mass index were the dominant contributors.

**Conclusion:**

The calibrated model provided moderate discrimination and favorable calibration for hospitalization-level blood culture positivity risk stratification in the pooled temporal validation cohort. Performance varied across age groups, and much of the predictive information was concentrated in age, PCT, and BMI. The model does not predict the index culture result alone or diagnose clinically adjudicated bloodstream infection. Multicenter external and prospective validation using fixed prediction horizons and adjudicated outcomes is required before clinical implementation.

## 1 Introduction

Bloodstream infection (BSI) remains a major clinical challenge because of its heterogeneous presentation and potential progression to sepsis, organ dysfunction, and death (1, 2). Blood culture remains the principal method for obtaining microbiological evidence, but pathogen identification and antimicrobial susceptibility testing require time (3–5). Among hospitalized patients who have already undergone blood culture collection, routinely available clinical information may therefore support interim risk stratification while microbiological results are pending. In the present study, the target was laboratory-recorded blood culture positivity from index collection to hospital discharge rather than the index culture result alone or clinically adjudicated BSI.

Procalcitonin (PCT), C-reactive protein (CRP), white blood cell count, neutrophil-related indices, and selected biochemical parameters are commonly used to assess the likelihood and severity of bacterial infection. Among these biomarkers, PCT has demonstrated diagnostic value for bacterial infection and bacteremia; however, in clinically heterogeneous populations, reliance on PCT alone remains insufficient for stable and accurate risk prediction (6, 7). Moreover, the interpretation of biomarkers may be influenced by age, comorbidities, inflammatory status, organ dysfunction, prior antimicrobial exposure, and physicians' selective ordering of laboratory tests. Therefore, reliance on a single biomarker is unlikely to capture the heterogeneous clinical characteristics associated with laboratory-recorded blood culture positivity.

Machine learning approaches provide opportunities to integrate demographic characteristics, hematological indices, inflammatory biomarkers, and biochemical parameters and to capture nonlinear relationships among predictors. Previous studies have used electronic health record and routine laboratory data to predict bacteremia, fungemia, or blood culture outcomes, suggesting that routinely collected clinical information may support infection-related risk stratification (8–10). Nevertheless, existing studies frequently rely on random data splitting, provide limited assessment of probability calibration, or lack temporal validation, decision-analytic evaluation, and transparent model interpretation. Prediction models should therefore be evaluated not only by discrimination but also by calibration, validation design, clinical applicability, and reporting transparency (11, 12).

In addition, the clinical applicability of machine learning models depends on their stable performance in real-world hospital data. In real-world electronic health record data, missing laboratory measurements, repeated visits by the same patient, and substantial heterogeneity of patient populations are common. Missingness in certain laboratory variables may not simply represent random data loss, but may instead reflect physicians' test-ordering patterns based on their clinical assessment of disease severity. Therefore, during prediction model development, missing data should be handled appropriately, information leakage should be avoided, machine learning models should be compared with simpler clinical or statistical models, and model robustness should be evaluated through subgroup and sensitivity analyses (11, 12).

Against this background, we developed and temporally validated a Platt-calibrated XGBoost model using routinely available demographic and laboratory data. The model was compared with PCT-only and regression-based approaches and evaluated for discrimination, calibration, threshold-specific performance, decision-analytic value, and SHAP-based interpretability. The intended use was hospitalization-level risk stratification among patients who had already undergone blood culture collection.

## 2 Materials and methods

### 2.1 Study design and data source

This retrospective prediction model development and temporal validation study used routinely collected electronic health record and laboratory information system data from the Affiliated Hospital of Southwest Medical University between January 1, 2022, and September 7, 2024. Extracted data included demographic characteristics, routine laboratory measurements, blood culture sampling information, and laboratory-recorded blood culture outcomes. The study was reported with reference to TRIPOD + AI, and methodological considerations were informed by PROBAST + AI (11, 12).

### 2.2 Study population and outcome definition

The unit of analysis was the blood culture-related hospitalization record, with the first blood culture collection during each hospitalization defined as the index event. Multiple hospitalizations from the same patient were permitted. Patient identifiers were used only for grouped cross-validation, assessment of cross-period overlap, and construction of the new-patient validation subset. Records were eligible if they were linked to a valid hospitalization, had an identifiable index blood-culture date and laboratory-recorded outcome, and contained clinical data suitable for predictor extraction. Records with missing or unrecognizable outcome information or identifiers/date information required for cohort construction were excluded. Because all eligible records within the prespecified study period were included, no formal *a priori* sample-size calculation was performed; the development cohort contained 2,661 outcome events for 28 prespecified model inputs.

The primary outcome was at least one laboratory-recorded positive blood culture between index collection and hospital discharge (outcome_bsi_admission_any_positive). The index-culture result (outcome_bsi_first_sample) was used only for auditing and sensitivity analysis and was not used as a predictor. Because the outcome could include positivity detected on subsequent cultures, it represents hospitalization-level blood culture positivity rather than the index culture result alone. The available data did not permit systematic adjudication of contamination or confirmation of true BSI (13).

### 2.3 Candidate predictors and data preprocessing

Candidate predictors were derived from structured electronic health record and laboratory information system data. The final main model included 14 clinical predictors: age, body mass index (BMI), sex, procalcitonin (PCT), white blood cell count (WBC), platelet count (PLT), neutrophil ratio (NEU_R), high-sensitivity C-reactive protein (hsCRP), serum sodium (Na), serum potassium (K), alanine aminotransferase (ALT), total bilirubin (TBIL), albumin (ALB), and prothrombin time (PT). Sex was encoded as sex_male in the model matrix.

For each hospitalization, predictors were obtained from the most recent demographic or laboratory information recorded before or on the index date. When multiple measurements of the same variable were available, the value closest to the index date was used. Laboratory measurements recorded after the index date and variables directly related to blood culture results or the outcome definition were excluded from the predictor set to avoid information leakage. Patient and hospitalization identifiers and the blood culture sampling date were used only for record linkage, grouped validation, temporal splitting, and data auditing, and were not included as model predictors.

Before model development, variables were harmonized for naming and data type, and continuous variables were screened using prespecified clinically plausible ranges. Nonconvertible or implausible values were treated as missing rather than excluding the corresponding records. Detailed variable definitions, units, cleaning rules, and outlier ranges are provided in Supplementary Tables 1, 2. Additional candidate variables, including erythrocyte sedimentation rate, lactate, HbA1c, anion gap, creatinine, and D-dimer, were excluded from the reported models because of high or near-complete missingness; detailed missingness and model inclusion status are provided in Supplementary Table 3.

**Table 1:** Baseline characteristics of the development and validation cohorts.

Characteristic	Development cohort (2022–2023)	Temporal validation cohort (2024)	New-patient temporal validation subset (2024)	Absolute SMD
Cohort composition				
Records, *n*	32,491	11,898	10,785	
Outcome-positive records, *n* (%)	2,661 (8.19%)	894 (7.51%)	820 (7.60%)	
Unique patients, *n*	29,488	11,211	10,266	
Unique admissions, *n*	32,491	11,898	10,785	
Demographic characteristics				
Age, years	10.00 (1.00–59.00)	8.00 (1.00–58.00)	8.00 (1.00–59.00)	0.040
Adult, ≥18 years, *n* (%)	15,022 (46.2%)	5,172 (43.5%)	4,799 (44.5%)	0.056
Pediatric, <18 years, *n* (%)	17,070 (52.5%)	6,576 (55.3%)	5,847 (54.2%)	0.055
Age missing, *n* (%)	399 (1.2%)	150 (1.3%)	139 (1.3%)	0.003
Male sex, *n* (%)	19,054 (58.6%)	6,975 (58.6%)	6,293 (58.3%)	0.004
BMI, kg/m^2^	17.86 (15.42–22.03)	17.78 (15.42–21.93)	18.08 (15.54–22.15)	0.003
Laboratory variables				
PCT, ng/mL	0.31 (0.12–1.13)	0.20 (0.06–0.76)	0.21 (0.06–0.79)	0.082
WBC, × 10⁹/L	9.40 (6.44–13.38)	9.21 (6.52–12.97)	9.33 (6.67–13.14)	0.022
PLT, × 10⁹/L	252 (181–332)	254 (183–339)	255 (185–339)	0.039
Neutrophil ratio, %	67.10 (46.90–81.30)	66.50 (46.00–81.50)	66.90 (46.20–81.80)	0.016
hsCRP, mg/L	7.20 (0.50–36.99)	12.15 (6.01–57.21)	11.94 (5.79–58.31)	0.182
Sodium, mmol/L	138.00 (135.60–140.50)	137.20 (135.00–139.60)	137.30 (135.00–139.70)	0.146
Potassium, mmol/L	4.18 (3.78–4.64)	4.16 (3.78–4.64)	4.16 (3.78–4.63)	0.015
ALT, U/L	21.50 (13.30–41.98)	23.10 (13.90–47.00)	23.30 (14.00–47.32)	0.020
TBIL, µmol/L	11.10 (7.20–18.90)	10.80 (7.40–19.00)	11.00 (7.50–19.30)	0.002
Albumin, g/L	35.40 (30.60–40.90)	33.60 (29.65–37.80)	33.60 (29.60–37.80)	0.297
PT, s	13.00 (11.90–14.50)	12.90 (11.90–14.20)	12.90 (11.90–14.20)	0.065
Selected predictor missingness				
ALT missing, *n* (%)	17,457 (53.73%)	7,624 (64.08%)	6,789 (62.95%)	0.212
TBIL missing, *n* (%)	17,461 (53.74%)	7,625 (64.09%)	6,789 (62.95%)	0.211
Albumin missing, *n* (%)	17,455 (53.72%)	7,623 (64.07%)	6,788 (62.94%)	0.211
PT missing, *n* (%)	18,622 (57.31%)	6,909 (58.07%)	6,135 (56.88%)	0.015

Values are presented as median (interquartile range) for continuous variables and *n* (%) for categorical variables unless otherwise indicated. Absolute standardized mean differences (SMDs) compare the 2022–2023 development cohort with the 2024 temporal validation cohort and are descriptive rather than inferential. Outcome-positive records refer to the prespecified hospitalization-level primary outcome.

Adult and pediatric categories do not sum to the total because of missing age values. Laboratory summary statistics were calculated from available observations.

**Table 2:** Model discrimination and calibration performance.

Model	Development ROC-AUC	Development PR-AUC	Development Brier score	Validation ROC-AUC (95% CI)	Validation PR-AUC	Validation Brier score	Validation calibration slope
PCT alone	0.662	0.202	0.0717	0.670 (0.650–0.691)	0.196	0.0670	1.093
Exploratory *post hoc* Age + PCT + BMI logistic	0.741	0.227	0.0694	0.757 (0.742–0.773)	0.216	0.0643	1.111
Conventional logistic (14 predictors)	0.753	0.244	0.0689	0.766 (0.751–0.782)	0.233	0.0638	1.071
Logistic + missingness indicators	0.766	0.256	0.0682	0.776 (0.760–0.791)	0.232	0.0636	1.019
LASSO logistic	0.761	0.244	0.0688	0.775 (0.760–0.790)	0.230	0.0638	1.044
XGBoost	0.782	0.271	0.0672	0.798 (0.784–0.813)	0.266	0.0619	1.061

Development-cohort values represent patient-grouped five-fold out-of-fold predictions after model-specific Platt calibration. Temporal-validation values are from the independent 2024 cohort. The Age + PCT + BMI logistic model was added as an exploratory *post hoc* comparator after SHAP analysis identified age, PCT, and BMI as the three dominant contributors. Its validation performance therefore should not be interpreted as independent validation of a prespecified three-predictor set. For the exploratory Age + PCT + BMI model, missing values were imputed using parameters estimated from the development cohort; the logistic model was fitted using development data only; and Platt calibration was fitted to patient-grouped development out-of-fold predictions before application to the 2024 cohort. In the 2024 temporal validation cohort, XGBoost exceeded logistic regression with missingness indicators by *Δ*ROC-AUC = 0.023 (95% CI 0.015–0.030; paired DeLong *P* < 0.001) and exceeded the exploratory Age + PCT + BMI model by *Δ*ROC-AUC = 0.041 (95% CI 0.031–0.051; paired DeLong *P* < 0.001), although the absolute improvements were modest.

Missing-value imputation was performed after temporal splitting. Continuous predictors were imputed using medians estimated from the entire 2022–2023 development cohort, whereas sex was imputed using the development-cohort modal value. These imputation parameters were fixed and then applied to the 2024 temporal validation cohort and the new-patient validation subset. The temporal validation cohort was not used to estimate imputation parameters or for model training, hyperparameter tuning, probability calibration, or threshold selection. Missingness indicators were retained as additional inputs in the primary model; their contribution was evaluated separately in sensitivity analyses.

### 2.4 Dataset splitting and validation strategy

To improve the temporal independence of model validation and to better simulate model performance in subsequent clinical data, this study adopted a time-based dataset splitting strategy rather than random splitting. The date variable used for temporal splitting was the blood culture sampling date (sample_date), defined as the sampling date of the first blood culture sample corresponding to the index record. Based on this date, data from January 1, 2022, to December 31, 2023, were defined as the model development set, whereas data from January 1, 2024, to September 7, 2024, were defined as the temporal validation set.

There was no overlap in hospitalization identifiers between the two cohorts. The temporal validation cohort was completely held out from the development and selection of the prespecified candidate models and the primary XGBoost model and was not used for estimating imputation parameters, model training, hyperparameter tuning, probability calibration, or threshold selection. The exploratory *post hoc* Age + PCT + BMI comparator was added after inspection of temporal-validation SHAP results, as described in Section 2.5.

Given that the same patient could have multiple hospitalizations across calendar periods, patient-level overlap between the development and temporal validation cohorts was assessed. A new-patient temporal validation subset was constructed by excluding all 2024 hospitalization records from patients whose identifiers appeared in the development cohort. Multiple eligible hospitalization records from the same patient during 2024 were retained. This subset was used only for sensitivity validation and did not participate in model development. Cohort sizes, event counts, and event rates are reported in Section 3.1.

Within the model development set, patient-grouped five-fold cross-validation was used for internal validation, model comparison, and hyperparameter tuning. Specifically, cross-validation was grouped by unique patient identifier so that all records from the same patient could appear only within the same cross-validation fold. This approach prevented records from the same patient from being simultaneously included in the training and validation folds, thereby addressing patient-level overlap during internal cross-validation. The internal cross-validation results were used primarily for model selection and stability assessment during the development stage, and were not considered the main evidence for final model generalizability.

Therefore, the validation strategy in this study consisted of three levels. First, patient-grouped five-fold cross-validation within the 2022–2023 development set was used to evaluate internal stability during model development. Second, the temporal validation set from January 1, 2024, to September 7, 2024, was used to evaluate model performance in a subsequent temporal population. Third, the 2024 new-patient validation set was used to further assess model robustness in patients who had not participated in the development stage. The primary validation results of this study were based on the 2024 temporal validation set.

### 2.5 Model development, calibration, and evaluation

Several prespecified candidate models were developed within the 2022–2023 development cohort: a PCT-only logistic regression model, conventional multivariable logistic regression using the 14 original clinical predictors, logistic regression with the 14 clinical predictors and 14 missingness indicators, LASSO logistic regression, and XGBoost. The 2024 temporal validation cohort was not used for training, hyperparameter tuning, calibration, threshold selection, or selection of the primary model. After SHAP analysis of the final XGBoost model in the 2024 temporal validation cohort identified age, PCT, and BMI as the three dominant contributors, an Age + PCT + BMI logistic model was added as an exploratory *post hoc* comparator. Because this predictor set was selected after inspection of the temporal-validation SHAP results, its performance in the 2024 cohort was not interpreted as independent validation of a prespecified three-predictor model.

LASSO tuning was performed using patient-grouped cross-validation within the development cohort, with the lambda.1se model reported as the primary LASSO model and lambda.min retained for supplementary analysis. XGBoost hyperparameters were tuned using patient-grouped five-fold cross-validation, with PR-AUC as the optimization metric. Class imbalance was addressed using scale_pos_weight, set to the development-cohort negative-to-positive ratio (11.210); no oversampling, undersampling, or SMOTE-based resampling was applied. Final XGBoost hyperparameters and tuning results are provided in Supplementary Table 4.

Platt scaling was applied separately to each prespecified candidate model and to the exploratory *post hoc* Age + PCT + BMI comparator. Calibration functions were fitted using patient-grouped out-of-fold predictions from the development cohort and fixed before application to the 2024 temporal validation cohort. For the Age + PCT + BMI model, missing predictor values were imputed using parameters estimated from the 2022–2023 development cohort only, the logistic coefficients were estimated using development data, and Platt calibration was fitted using development-cohort out-of-fold predictions. The 2024 temporal validation cohort was not used to estimate its imputation parameters, logistic coefficients, or calibration parameters. The corresponding coefficients and Platt calibration parameters are reported in Supplementary Table 8. For XGBoost, Platt scaling was prespecified as the primary calibration method, while isotonic regression was evaluated as a supplementary sensitivity analysis. Because class weighting altered the scale of raw XGBoost outputs, all threshold-specific classification and decision-curve analyses were based on Platt-calibrated probabilities.

Model performance was evaluated using discrimination, calibration, threshold-specific classification measures, and exploratory decision-curve analysis. Discrimination was assessed using ROC-AUC and PR-AUC. Calibration was assessed using the Brier score, log loss, calibration intercept and slope, observed-to-predicted ratio, and agreement between mean predicted risk and observed event rate.

Threshold-specific performance was evaluated at probabilities of 5%, 7.5%, 10%, 15%, and 20% using sensitivity, specificity, positive predictive value, negative predictive value, F1 score, predicted-positive proportion, confusion-matrix measures, and number needed to flag (NNF). NNF was defined as the number of model-positive records divided by the number of true-positive records, equivalent to 1/PPV. These thresholds were used to characterize alternative classification trade-offs and were not considered clinically validated diagnostic or treatment thresholds.

Exploratory decision-curve analysis was performed across threshold probabilities from 1% to 50%, with primary interpretation focused on the prespecified 5%–20% range (14). A model-positive classification represented a hypothetical risk alert that could prompt additional clinical reassessment or monitoring. The conventional ‘treat-all’ and ‘treat-none’ strategies were used only in their decision-curve sense and did not represent initiation or withholding of antimicrobial therapy. Because no model-triggered intervention was prospectively evaluated, decision-curve results were interpreted as exploratory rather than evidence of clinical effectiveness.

### 2.6 Model interpretability, subgroup analyses, and sensitivity analyses

SHAP (SHapley Additive exPlanations) was used to interpret prediction patterns of the final XGBoost model (15). SHAP values were calculated for the underlying XGBoost model before Platt calibration and were summarized using mean absolute SHAP values and a SHAP summary plot. SHAP analyses were used to describe predictor contributions to model output rather than to infer causal relationships.

Prespecified age-stratified analyses evaluated the fixed final Platt-calibrated XGBoost model separately in adults (≥18 years) and children (<18 years) within the 2024 temporal validation cohort. To quantify the contribution of age-group separation to pooled discrimination, additional analyses were performed among validation records with known age. Two age-only logistic models were evaluated: one using age as a continuous predictor and the other using a binary adult indicator (≥18 years). Both models were fitted in the 2022–2023 development cohort using records with known age and then applied without refitting to the 2024 temporal validation cohort. These models and the primary XGBoost model were evaluated in the same 11,748 known-age validation records. A pair-count-weighted pooled within-age-stratum ROC-AUC was additionally calculated by combining discrimination within the adult and pediatric strata while excluding between-age-group case-control comparisons. An equal-weight adult/pediatric standardized AUC was examined as a supplementary sensitivity analysis. Records with missing age were described and evaluated separately.

Sensitivity analyses included an XGBoost model without missingness indicators and three predictor-removal models: (1) without age, (2) without age and all missingness indicators, and (3) without both age and BMI together with their corresponding missingness indicators (age_missing and BMI_missing). Each sensitivity model was retrained and Platt-calibrated using the same patient-grouped development workflow as the primary model. The no-age model trained in the overall development cohort was additionally evaluated separately within the adult and pediatric validation subgroups. These subgroup evaluations were distinct from the separately retrained adult-only and pediatric-only XGBoost models, which were evaluated as exploratory analyses. A new-patient temporal validation subset was also evaluated after excluding 2024 records from patients whose identifiers had appeared in the 2022–2023 development cohort.

All analyses were performed using R version 4.5.3. Ninety-five percent confidence intervals for ROC-AUC were estimated using DeLong's method. In the 2024 temporal validation cohort, paired DeLong tests compared the ROC-AUC of the final XGBoost model with those of logistic regression with missingness indicators and the exploratory *post hoc* Age + PCT + BMI logistic model. AUC differences were calculated as XGBoost minus the corresponding comparator, with 95% confidence intervals reported. For the index-culture-positive sensitivity outcome, the difference in ROC-AUC relative to the primary hospitalization-level outcome was quantified using a patient-cluster bootstrap 95% confidence interval. Other model comparisons were interpreted descriptively.

### 2.7 Ethics statement

This study was approved by the Ethics Committee of the Affiliated Hospital of Southwest Medical University (approval number: KY2025729). Because this was a retrospective study based on existing clinical care data and all data were de-identified before analysis, the requirement for informed consent was waived by the ethics committee. All study procedures were conducted in accordance with the Declaration of Helsinki and relevant ethical standards for medical research. Data processing and analysis were performed in strict compliance with requirements for patient privacy protection.

## 3 Results

### 3.1 Study population and baseline characteristics

The study flow and dataset splitting are shown in Figure 1. Among 44,440 blood culture-related hospitalization records identified between January 1, 2022, and September 7, 2024, 51 were excluded because of missing or unrecognizable outcome information, leaving 44,389 records for analysis. Overall, 3,555 records (8.01%) met the hospitalization-level outcome of at least one laboratory-recorded positive blood culture. Of these, 3,088 (86.86%) were positive at the index culture and 467 (13.14%) had a negative index culture followed by subsequent positivity during the same hospitalization. Repeated blood culture testing occurred in 5,559 records (12.52%), with outcome rates of 29.72% among records with repeated testing and 4.90% among those with a single culture collection.

**Figure 1:**
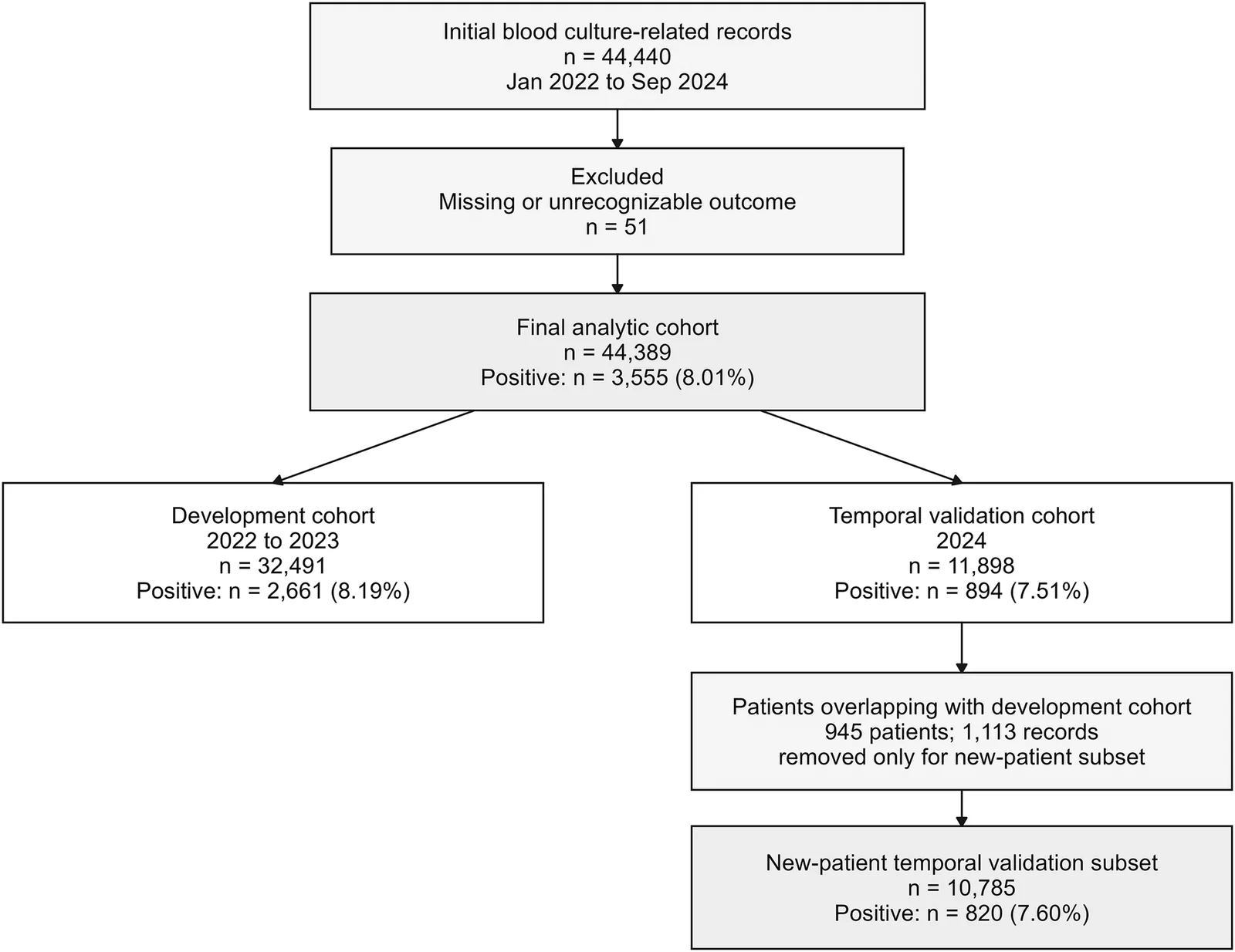
Study flow and temporal dataset splitting. A total of 44,440 blood culture-related hospitalization records were screened from January 2022 to September 2024. After excluding 51 records with missing or unrecognizable outcome information, 44,389 records were included in the final analytic cohort. Records from 2022 to 2023 were used as the development cohort, whereas records from 2024 were reserved for temporal validation. The new-patient temporal validation subset was derived from the 2024 temporal validation cohort by excluding patients who had appeared in the development cohort. ‘Positive’ refers to laboratory-recorded in-hospital blood culture positivity, rather than manually adjudicated true bloodstream infection.

The 2022–2023 development cohort included 32,491 hospitalization records with 2,661 outcome-positive records (8.19%), whereas the 2024 temporal validation cohort included 11,898 records with 894 outcome-positive records (7.51%). After excluding 2024 records from patients who had appeared in the development cohort, the new-patient temporal validation subset comprised 10,785 records with 820 outcome-positive records (7.60%). Baseline demographic and laboratory characteristics are summarized in Table 1. The temporal validation cohort included 5,172 adult records (43.47%) and 6,576 pediatric records (55.27%), with hospitalization-level blood culture positivity rates of 14.29% and 1.99%, respectively, indicating substantial differences in baseline risk between age groups.

Several laboratory variables showed modest temporal differences, including lower median PCT and albumin and higher hsCRP in the temporal validation cohort, whereas other routinely measured variables were broadly similar between cohorts. Predictor missingness varied substantially and is detailed in Table 1 and Supplementary Table 3.

Admission-ward composition is summarized in Supplementary Table 7. In the development and 2024 temporal validation cohorts, neonatology admissions accounted for 13.64% (4,431/32,491) and 12.59% (1,498/11,898), respectively; oncology admissions accounted for 1.38% (447/32,491) and 1.54% (183/11,898), respectively; and records explicitly coded as ICU/PICU at admission accounted for 0.01% (4/32,491) and 0.02% (2/11,898), respectively. Pulmonary and Critical Care Medicine accounted for an additional 5.34% and 4.23% of admissions, respectively, and was retained as a separate admission-ward category rather than being classified as ICU/PICU. Importantly, the available ward field represented the ward at hospital admission and did not necessarily reflect the patient's ward or ICU status at the time of index blood-culture collection.

### 3.2 Model performance and calibration

Model performance is summarized in Table 2 and Figure 2. In development-cohort out-of-fold predictions, XGBoost achieved a ROC-AUC of 0.782 and a PR-AUC of 0.271. In the 2024 temporal validation cohort, the Platt-calibrated XGBoost model achieved a ROC-AUC of 0.798 (95% CI: 0.784–0.813) and a PR-AUC of 0.266, compared with ROC-AUCs of 0.776 (95% CI: 0.760–0.791) for logistic regression with missingness indicators and 0.757 (95% CI: 0.742–0.773) for the exploratory *post hoc* Age + PCT + BMI logistic comparator. Performance of the remaining comparator models is reported in Table 2.

**Figure 2:**
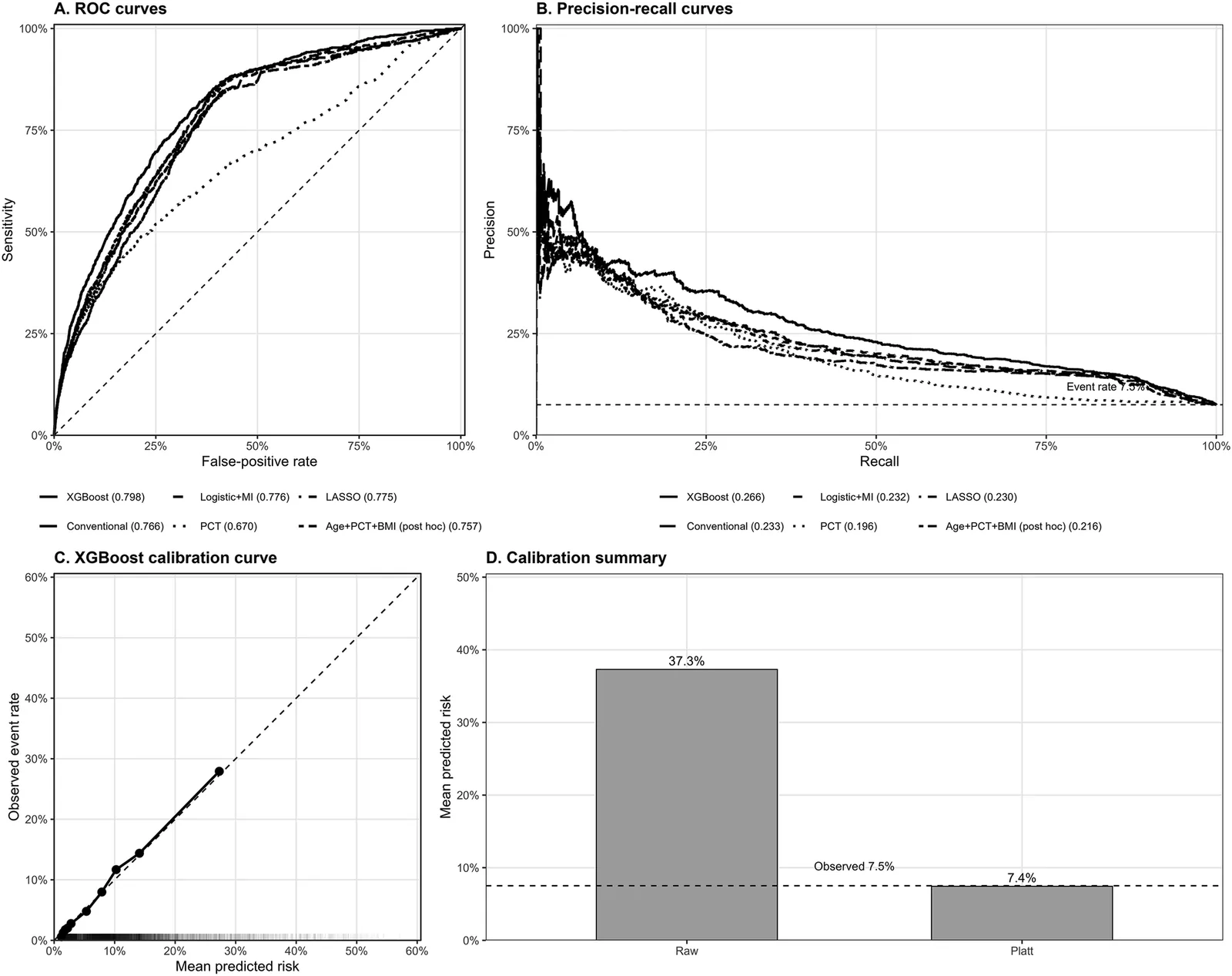
Discrimination and calibration of selected models in the 2024 temporal validation cohort. **(A)** Receiver operating characteristic curves. **(B)** Precision-recall curves, with the dashed horizontal line indicating the observed event rate. **(C)** Calibration curve of the Platt-calibrated XGBoost model. The diagonal dashed line represents perfect calibration, and rug marks along the *x*-axis show the distribution of individual predicted probabilities. The displayed probability range was limited to 0–0.60 to improve visualization of the region containing the large majority of predictions. **(D)** Comparison of mean predicted risk before and after Platt calibration with the observed event rate. The Platt-calibrated XGBoost model was selected as the final model.

Raw XGBoost probabilities substantially overestimated absolute risk, with a mean predicted risk of 37.30% compared with an observed event rate of 7.51%. After Platt calibration, discrimination was unchanged, whereas mean predicted risk decreased to 7.44%, the observed-to-predicted ratio was 1.01, the Brier score was 0.0619, and the calibration slope was 1.061. Supplementary isotonic-calibration results are provided in Supplementary Table 5. The Platt-calibrated XGBoost model was retained as the final main model.

In paired comparisons within the temporal validation cohort, XGBoost showed a statistically significant but modest increase in ROC-AUC compared with logistic regression with missingness indicators (*Δ*ROC-AUC = 0.023, 95% CI: 0.015–0.030; paired DeLong test, *P* < 0.001). Compared with the exploratory *post hoc* Age + PCT + BMI logistic comparator, the corresponding difference was 0.041 (95% CI: 0.031–0.051; paired DeLong test, *P* < 0.001). All subsequent threshold-specific, subgroup, and sensitivity analyses used probabilities from the Platt-calibrated XGBoost model.

### 3.3 Classification performance at different thresholds and exploratory decision curve analysis

Threshold-specific performance of the final Platt-calibrated XGBoost model is summarized in Table 3 and Figure 3. At the 5% threshold, 5,512 records (46.3%) were classified as model-positive, with a sensitivity of 0.877, specificity of 0.570, positive predictive value of 0.142, negative predictive value of 0.983, and number needed to flag of 7.03. The high negative predictive value should be interpreted in the context of the low event rate and does not establish that clinically adjudicated BSI can be safely excluded.

**Table 3:** Threshold-specific classification performance of the platt-calibrated XGBoost model in the 2024 temporal validation cohort.

Threshold probability	N	Event rate	Model-positive, n (%)	TP	FP	TN	FN	Sensitivity	Specificity	PPV	NPV	Number needed to flag	Net benefit
5.0%	11,898	7.51%	5,512 (46.3%)	784	4,728	6,276	110	0.877	0.570	0.142	0.983	7.03	0.0450
7.5%	11,898	7.51%	4,404 (37.0%)	713	3,691	7,313	181	0.798	0.665	0.162	0.976	6.18	0.0348
10.0%	11,898	7.51%	3,019 (25.4%)	577	2,442	8,562	317	0.645	0.778	0.191	0.964	5.23	0.0257
15.0%	11,898	7.51%	1,493 (12.5%)	379	1,114	9,890	515	0.424	0.899	0.254	0.951	3.94	0.0153
20.0%	11,898	7.51%	876 (7.4%)	274	602	10,402	620	0.306	0.945	0.313	0.944	3.20	0.0104

Classification performance is shown for the final Platt-calibrated XGBoost model in the 2024 temporal validation cohort. Number needed to flag (NNF) was calculated as the number of model-positive records divided by the number of true-positive records, equivalent to 1/PPV. Net benefit was calculated at each threshold probability using the standard decision-curve formulation. TP, true positive; FP, false positive; TN, true negative; FN, false negative; PPV, positive predictive value; NPV, negative predictive value; NNF, number needed to flag.

**Figure 3:**
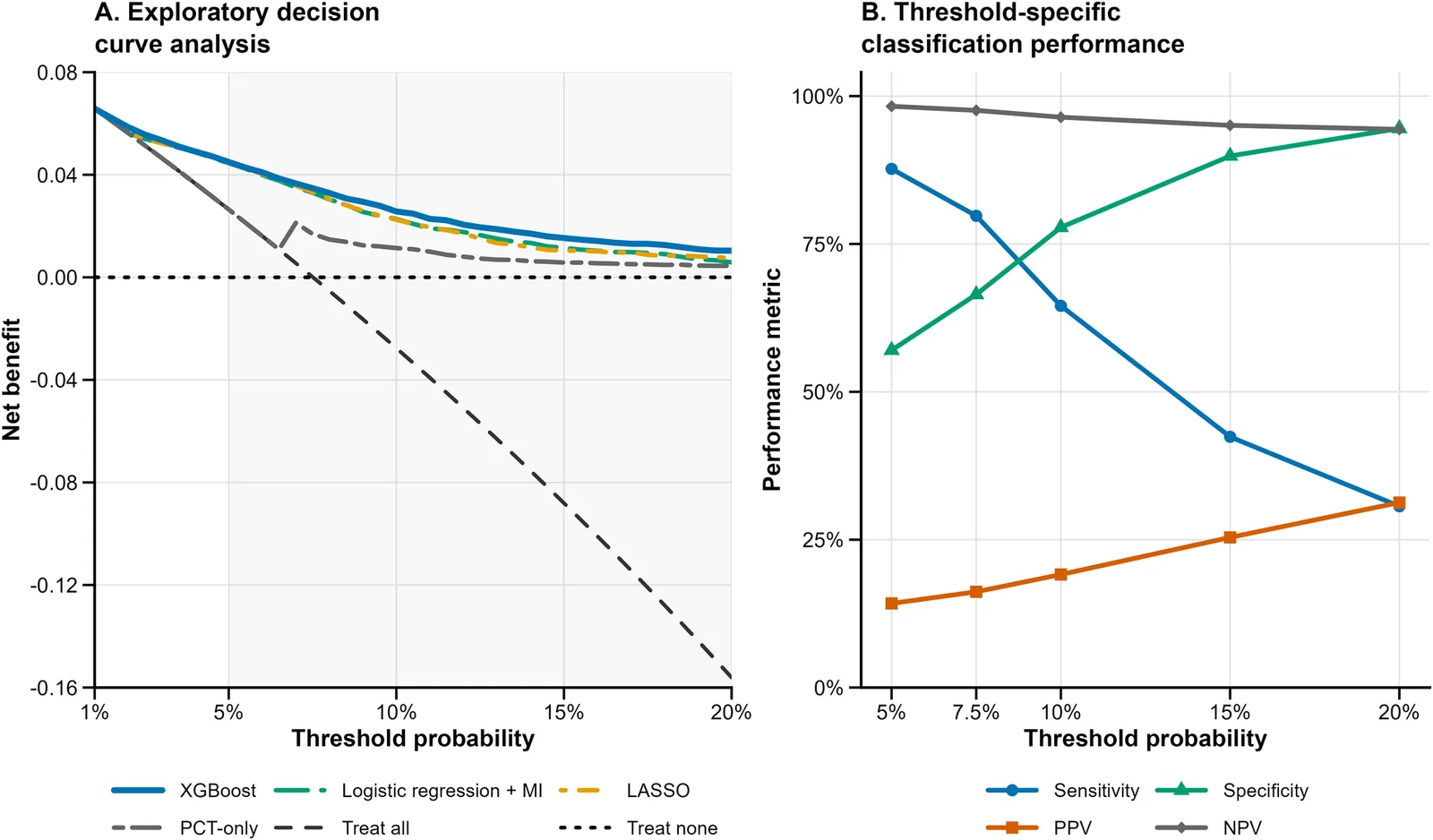
Exploratory decision curve analysis and threshold-specific classification performance of the final model. **(A)** Exploratory decision curve analysis of the Platt-calibrated XGBoost model and comparator models in the 2024 temporal validation cohort. All model curves were based on probabilities obtained after model-specific Platt calibration. A model-positive classification represented a hypothetical risk alert that could prompt additional clinical reassessment or closer monitoring. The conventional ‘treat-all’ and ‘treat-none’ labels were used in their decision-curve sense and did not refer specifically to antimicrobial treatment. The shaded area indicates the prespecified analysis range of 5% to 20%. **(B)** Sensitivity, specificity, positive predictive value, and negative predictive value at prespecified probability thresholds. These thresholds were used to describe alternative classification trade-offs and were not validated as diagnostic or treatment decision cutoffs. MI, missingness indicators; NPV, negative predictive value; PCT, procalcitonin; PPV, positive predictive value; XGBoost, extreme gradient boosting.

At the 7.5% threshold, sensitivity decreased to 0.798 while specificity increased to 0.665. With further increases in threshold, the proportion of model-positive records and sensitivity decreased, whereas specificity and positive predictive value increased; detailed metrics for the 7.5%, 10%, 15%, and 20% thresholds are provided in Table 3. These thresholds were used to characterize classification trade-offs and were not considered clinically validated diagnostic or treatment cutoffs.

Exploratory decision curve analysis showed positive calculated net benefit for the final model relative to the conventional ‘treat-all’ and ‘treat-none’ strategies across the prespecified 5%–20% threshold range. Mean net benefit over this range was 0.0227 for XGBoost and 0.0098 for the PCT-only model. Because the analysis assumed a hypothetical risk-alert action and no model-triggered intervention was prospectively evaluated, these findings should be interpreted as exploratory decision-analytic evidence rather than evidence of improved clinical outcomes.

### 3.4 Model interpretability analysis

SHAP-based interpretation of the final XGBoost model is shown in Figure 4. In the 2024 temporal validation cohort, age accounted for 42.87% of the total mean absolute SHAP value, followed by PCT at 22.67% and BMI at 6.07%; together, these three predictors accounted for 71.61% of total SHAP importance. Missingness indicators collectively accounted for 12.20%, with additional contributions from hematological, inflammatory, biochemical, and coagulation-related variables.

**Figure 4:**
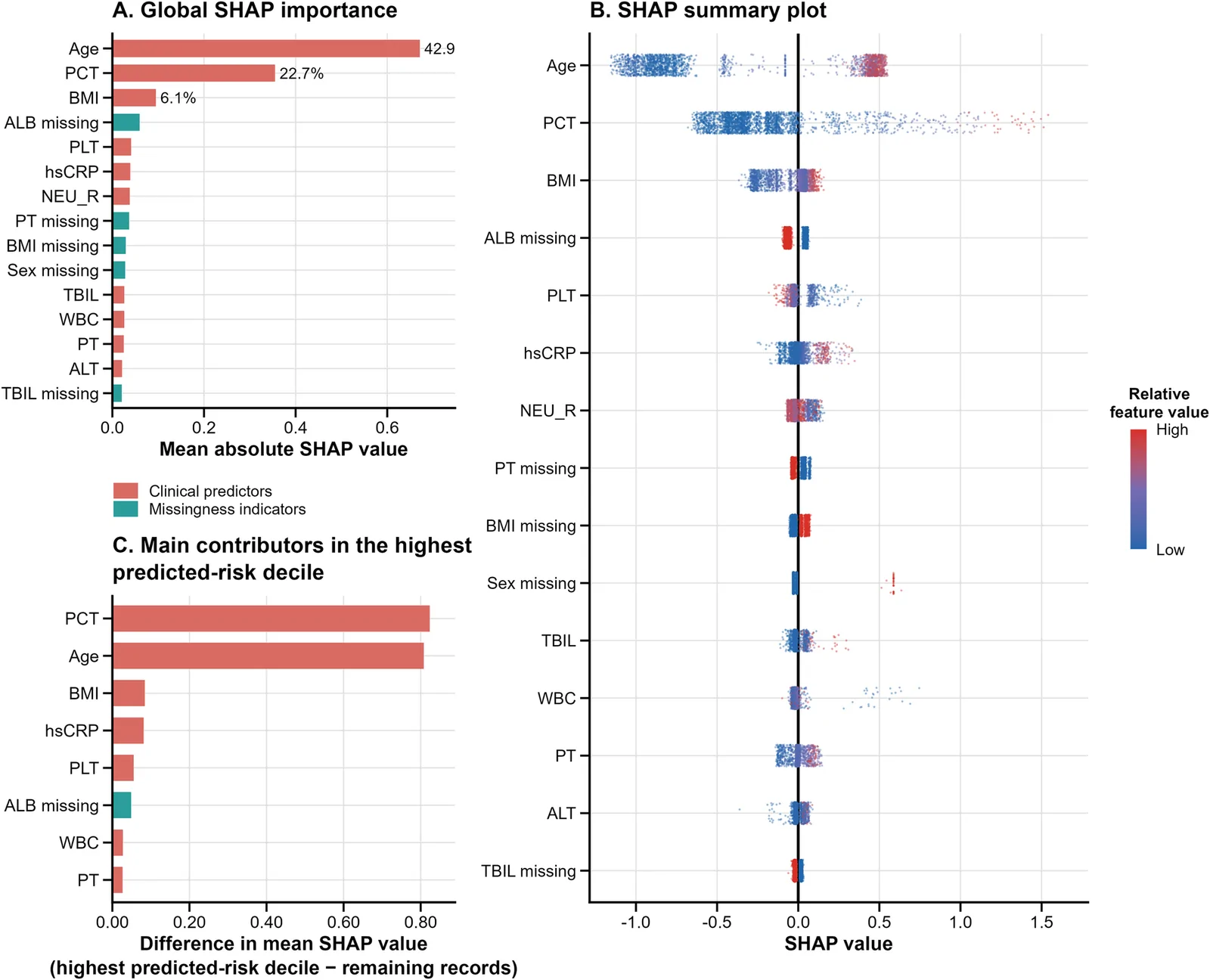
SHAP-based interpretation of the final blood culture positivity risk stratification model**.** SHAP values were calculated for the underlying XGBoost model before Platt probability calibration and were used to interpret contributions to the model output rather than calibrated probabilities. **(A)** Global SHAP importance of the leading predictors in the 2024 temporal validation cohort. Bar length represents the mean absolute SHAP value. Percentages displayed for the three leading predictors indicate their relative contributions to the total mean absolute SHAP value. Clinical predictors and missingness indicators are shown separately. **(B)** SHAP summary plot showing the magnitude and direction of predictor contributions to the underlying XGBoost model output. Positive SHAP values indicate an increase in model output, whereas negative values indicate a decrease. For visualization, up to 2,500 observations per predictor were randomly sampled using a fixed seed. Continuous feature values were winsorized at the 1st and 99th percentiles and rescaled from 0 to 1 within each predictor. Blue and red therefore represent relatively lower and higher values, respectively, within the same predictor. Binary missingness indicators retained values of 0 and 1. **(C)** Main contributors among records in the highest predicted-risk decile, quantified as the difference in mean SHAP value between the highest predicted-risk decile and the remaining records. These results describe contributions to model output and should not be interpreted as causal effects. ALB, albumin; BMI, body mass index; hsCRP, high-sensitivity C-reactive protein; NEU_R, neutrophil ratio; PCT, procalcitonin; PLT, platelet count; PT, prothrombin time; SHAP, SHapley Additive explanations; TBIL, total bilirubin; WBC, white blood cell count; XGBoost, extreme gradient boosting.

The sex-missing indicator was present in 150 of 11,898 records (1.26%), of which 24 were outcome-positive (event rate, 16.0%). All 150 records with missing sex also had missing age, representing complete overlap with the age-missing subset; no records had isolated missing sex or isolated missing age.

The SHAP summary plot showed that higher age and PCT were generally associated with higher underlying model output, while other predictors showed smaller and more heterogeneous contributions. Age and PCT also showed the largest positive SHAP contributions among records in the highest predicted-risk decile. These patterns describe contributions to model output rather than causal relationships.

### 3.5 Subgroup and sensitivity analyses

The results of subgroup and sensitivity analyses are summarized in Table 4 and Supplementary Table 6. Among the 11,748 temporal-validation records with known age, the primary XGBoost model achieved a ROC-AUC of 0.800 (95% CI: 0.785–0.815). In the same records, the continuous age-only logistic model and adult-indicator-only logistic model each achieved a ROC-AUC of 0.721 (95% CI: 0.705–0.738 and 0.708–0.734, respectively). When discrimination was restricted to comparisons within the adult and pediatric strata, the pair-count-weighted pooled within-age-stratum ROC-AUC was 0.676 (95% CI: 0.656–0.696). Consistent with this result, discrimination of the fixed primary XGBoost model was substantially lower when evaluated separately in adults and children, with ROC-AUCs of 0.679 (95% CI: 0.657–0.701) and 0.663 (95% CI: 0.616–0.710), respectively. These findings indicate that between-age-group differences contributed materially to the higher pooled-cohort discrimination.

**Table 4:** Subgroup, age-mix, new-patient, and sensitivity analyses in the 2024 temporal validation cohort.

Analysis	Validation set/subgroup	Model	Events/N	Event rate	Mean risk	O/E ratio	ROC-AUC (95% CI)	PR-AUC	Brier score	Calibration slope
Reference validation	Overall 2024 temporal validation cohort	Primary Platt-calibrated XGBoost	894/11,898	7.51%	7.44%	1.01	0.798 (0.784–0.813)	0.266	0.0619	1.061
Known-age comparison	Known-age validation records	Primary Platt-calibrated XGBoost	870/11,748	7.41%	7.35%	1.01	0.800 (0.785–0.815)	0.267	0.0610	1.061
Known-age comparison	Known-age validation records	Age-only continuous logistic	870/11,748	7.41%	7.89%	0.94	0.721 (0.705–0.738)	0.140	0.0655	1.120
Known-age comparison	Known-age validation records	Adult-indicator-only logistic	870/11,748	7.41%	7.76%	0.95	0.721 (0.708–0.734)	0.136	0.0649	1.123
Age subgroup	Adult subgroup, age ≥18 years	Primary Platt-calibrated XGBoost	739/5,172	14.29%	13.77%	1.04	0.679 (0.657–0.701)	0.293	0.1141	0.996
Age subgroup	Pediatric subgroup, age <18 years	Primary Platt-calibrated XGBoost	131/6,576	1.99%	2.30%	0.87	0.663 (0.616–0.710)	0.061	0.0192	1.118
Within-age discrimination	Known-age validation records	Pair-count-weighted pooled within-age-stratum AUC	870/11,748				0.676 (0.656–0.696)			
Missing-age subgroup	Age missing	Primary Platt-calibrated XGBoost	24/150	16.00%	14.33%	1.12	0.492 (0.467–0.517)	0.148	0.1364	−0.701
New-patient validation	New-patient temporal validation subset	Primary Platt-calibrated XGBoost	820/10,785	7.60%	7.60%	1.00	0.791 (0.775–0.806)	0.257	0.0630	1.033
Sensitivity model	Overall 2024 temporal validation cohort	XGBoost without missingness indicators	894/11,898	7.51%	7.40%	1.02	0.797 (0.782–0.811)	0.270	0.0619	1.065
Sensitivity outcome	Overall 2024 temporal validation cohort	Primary XGBoost predictions; index-culture-positive outcome	771/11,898	6.48%	7.44%	0.87	0.795 (0.779–0.811)	0.241	0.0548	1.049

The reference model was the fixed primary Platt-calibrated XGBoost model. Known-age comparisons excluded the 150 validation records with missing age so that the primary model, continuous age-only model, and adult-indicator-only model were evaluated in exactly the same 11,748 records. The pair-count-weighted pooled within-age-stratum AUC combines adult and pediatric within-group AUCs weighted by the number of comparable case-control pairs and excludes between-age-group comparisons. For the 150 records with missing age, 24 were outcome-positive (16.0%); discrimination was poor and should be interpreted cautiously because of the small subgroup size. For the index-culture-positive sensitivity outcome, the difference versus the primary outcome was ΔROC-AUC = 0.0036 (patient-cluster bootstrap 95% CI −0.0018 to 0.0088).

Age was missing in 150 validation records, of which 24 were outcome-positive (event rate, 16.0%). All 150 age values were originally missing or blank; none resulted from application of the prespecified 0–120-year plausibility-range cleaning rule. The specific reasons for these originally missing or blank age values could not be determined from the available source data. All 41 out-of-range age values identified in the full analytic cohort occurred in the development cohort. In the age-missing validation subgroup, the primary XGBoost model showed poor discrimination, with a ROC-AUC of 0.492 (95% CI: 0.467–0.517); this estimate should be interpreted cautiously given the small subgroup size.

Performance remained broadly similar in the new-patient validation subset (ROC-AUC 0.791) and after removal of all missingness indicators (ROC-AUC 0.797). When the outcome was restricted to index-culture positivity, the ROC-AUC was 0.795, corresponding to a difference of 0.0036 from the primary hospitalization-level outcome (patient-cluster bootstrap 95% CI: −0.0018 to 0.0088). Detailed calibration and other performance metrics for these analyses are provided in Table 4.

Predictor-removal analyses showed a pooled-cohort ROC-AUC of 0.788 after removal of age. When the same no-age model was evaluated separately within age strata, ROC-AUCs were 0.673 (95% CI: 0.651–0.695) in adults and 0.657 (95% CI: 0.606–0.709) in children. Removal of age together with all missingness indicators yielded a pooled ROC-AUC of 0.785, whereas removal of both age and BMI together with their corresponding missingness indicators reduced the ROC-AUC to 0.773 (95% CI: 0.757–0.789). Residual discrimination in the pooled cohort should not be interpreted as evidence of age-independent prediction because other laboratory variables and patterns of test availability may also encode age-related information. Separately retrained adult-only and pediatric-only models did not clearly improve within-group discrimination; detailed results are provided in Supplementary Table 6.

## 4 Discussion

In this single-center retrospective study, we developed and temporally validated a Platt-calibrated XGBoost model for hospitalization-level blood culture positivity using routinely available clinical information. The model showed moderate discrimination and favorable calibration in the pooled 2024 temporal validation cohort, with only modest improvement over regression-based comparators. However, discrimination was substantially lower within adult and pediatric subgroups, and age, PCT, and BMI accounted for most of the model's predictive contribution. These findings support interpretation of the model as an exploratory risk-stratification tool rather than a diagnostic model for clinically adjudicated bloodstream infection or a replacement for clinical decision-making.

Risk assessment before definitive microbiological results become available remains challenging because individual biomarkers do not fully capture the heterogeneity associated with blood culture positivity. PCT has shown value for predicting bacteremia or blood culture positivity, although its performance varies across populations (6, 7, 16). Consistent with this, the PCT-only model showed lower discrimination than the multivariable models in the present study. Previous studies have applied machine learning and regression approaches to blood culture-related prediction in hospitalized, emergency-department, and intensive-care populations (8–10, 16–20). The present study extends this literature through temporal validation, patient-grouped cross-validation, probability calibration, and model interpretation. However, its prediction target was laboratory-recorded blood culture positivity from index collection to hospital discharge rather than clinically adjudicated BSI, and the findings should be interpreted within this outcome definition.

Probability calibration was necessary because class weighting substantially inflated raw XGBoost probabilities. Platt calibration preserved discrimination while improving agreement between predicted and observed risk. Nevertheless, threshold-based performance requires cautious interpretation: lower thresholds increased sensitivity but generated substantial alert burden, whereas positive predictive values remained limited. Exploratory decision curve analysis showed positive calculated net benefit across the prespecified threshold range (14), but no model-triggered clinical intervention was prospectively evaluated. These findings therefore support further evaluation of risk-based reassessment or monitoring rather than independent antimicrobial or diagnostic decision-making.

SHAP analysis (15) showed that age, PCT, and BMI were the dominant contributors to model output. An exploratory *post hoc* logistic comparator constructed from these three SHAP-ranked predictors achieved a ROC-AUC of 0.757 in the temporal validation cohort. Because the predictor set was selected after inspection of the temporal-validation SHAP results, this performance should not be interpreted as independent validation of a prespecified three-predictor model. The modest incremental discrimination of XGBoost over simpler regression-based models may reflect nonlinear relationships and interactions rather than substantial additional predictive information. SHAP values describe contributions to model output rather than causal effects and should therefore be interpreted as explanations of model behavior rather than evidence of biological or clinical mechanisms.

Missingness requires variable-specific interpretation in real-world electronic health record data. Missing laboratory measurements may partly reflect test-ordering patterns, whereas missing demographic information is more likely to reflect registration or data-quality processes. In the 2024 temporal validation cohort, sex was missing in 150 records (1.26%), including 24 outcome-positive records (event rate, 16.0%); all 150 records also had missing age. Given this complete overlap and the small subset size, the relatively high SHAP contribution of the sex-missing indicator is more plausibly interpreted as reflecting a shared registration or data-quality pattern than a clinically meaningful test-ordering signal. Removal of all missingness indicators produced little change in overall model performance, although missingness mechanisms and data-registration practices should be reassessed before application in other healthcare settings.

The substantially lower discrimination within adult and pediatric subgroups indicates that pooled-cohort performance should not be interpreted as uniformly strong within-group risk ranking. This interpretation was reinforced by the pair-count-weighted pooled within-age-stratum ROC-AUC of 0.676, compared with 0.800 when the primary model was evaluated across all known-age validation records. The continuous age-only and adult-indicator-only models each achieved a ROC-AUC of 0.721, further demonstrating the contribution of separation between age groups to pooled discrimination. Nevertheless, removal of age from the XGBoost model did not isolate age-independent predictive information: although the pooled ROC-AUC remained 0.788, the same no-age model achieved ROC-AUCs of only 0.673 in adults and 0.657 in children. Other laboratory variables and patterns of test availability may also encode age-related information. Separately retrained adult-only and pediatric-only models did not clearly improve within-group discrimination. These findings support cautious interpretation of pooled performance and indicate that age-specific model updating, calibration, and external validation would be required before application to specific age populations.

This study has several strengths. It used a relatively large real-world cohort with temporal validation, patient-grouped cross-validation, and a new-patient validation subset to assess robustness under temporal separation and potential cross-period patient overlap. Model evaluation incorporated discrimination, calibration, threshold-based classification, exploratory decision analysis, and model interpretation. In addition, the primary XGBoost model was fully specified without using the 2024 temporal validation cohort for model training, hyperparameter tuning, probability calibration, threshold selection, or model selection.

This study has several limitations. First, this was a single-center retrospective study, and temporal validation and the new-patient validation subset do not constitute external validation. Generalizability to other institutions, populations, and laboratory workflows therefore remains uncertain. Second, the primary outcome covered the interval from index culture collection to hospital discharge and therefore had a variable prediction horizon. Patients with longer hospitalization or repeated cultures had greater opportunity for outcome detection, so the model should be interpreted as estimating hospitalization-level positivity rather than the index-culture result. Third, the laboratory-based outcome could not systematically distinguish blood culture contamination from true bloodstream infection (21). Future studies should incorporate contamination rules, repeated culture results, clinical evidence of infection, antimicrobial exposure, and expert adjudication. Fourth, blood culture testing is subject to indication bias because all included patients had already undergone culture collection. The model should therefore not be generalized to all hospitalized patients or used to determine whether blood cultures should be obtained. Fifth, several predictors had substantial missingness. Although missingness indicators and sensitivity analyses were used, missingness mechanisms and test-ordering or registration practices may differ across institutions and should be reassessed before external application. Sixth, imputation parameters were estimated from the entire development cohort rather than separately within each internal cross-validation training fold, which may have introduced some optimism into development-cohort out-of-fold estimates and calibration fitting. The 2024 temporal validation cohort, however, remained fully held out from imputation estimation, model training, tuning, calibration, and threshold selection. Seventh, predictor availability was established at the date level, and the exact sequence of laboratory testing and blood culture collection within the same day could not always be determined. Prospective studies using precise timestamps are needed to confirm real-time predictor availability. Eighth, several clinically important predictors were unavailable, including vital signs, comorbidities, immunosuppression, antimicrobial exposure, infection site, indwelling vascular catheters, other invasive procedures, ward/ICU status, and longitudinal laboratory changes. The observed discrimination therefore reflects the information available in the present demographic and laboratory dataset rather than a comprehensive bedside prediction model. Before clinical adoption is considered, these omitted variables should be incorporated and their incremental predictive value and the resulting model performance validated in independent and prospective populations. Ninth, discrimination was substantially lower within adult and pediatric subgroups than in the pooled cohort. The model should therefore not be assumed to perform equivalently across age groups, and age-specific updating and independent validation would be required before subgroup-specific application.

Future work should prioritize multicenter external and prospective validation, incorporation of clinically important variables unavailable in the current dataset, and evaluation across distinct age groups and clinical settings. Clinical impact studies are also needed before implementation to determine whether a predefined model-triggered action improves care without excessive alert burden. Until such evidence is available, the present model should be regarded as an exploratory hospitalization-level risk stratification model rather than an independent diagnostic or treatment decision system.

## 5 Conclusion

This study developed and temporally validated a Platt-calibrated XGBoost model that used routine clinical information available at initial blood culture collection to estimate the probability of any laboratory-recorded positive blood culture before hospital discharge. The model showed moderate discrimination and favorable calibration in the pooled 2024 temporal validation cohort, although discrimination was substantially lower within adult and pediatric subgroups.

The model estimates a hospitalization-level outcome that includes both index and subsequent blood culture positivity. It does not specifically predict the result of the index culture, distinguish contamination from true infection, diagnose clinically adjudicated BSI, determine whether blood cultures should be obtained, or independently guide antimicrobial treatment. Multicenter external validation, prospective evaluation, fixed prediction horizons, and clinically adjudicated outcomes are required before clinical implementation.

## Data Availability

The original contributions presented in the study are included in the article/Supplementary Material, further inquiries can be directed to the corresponding authors.
