# Supplementary material for: Development and temporal validation of a calibrated machine learning model for in-hospital blood culture positivity using routine clinical data
Source: Front Med (Lausanne). 2026 Oct 9;13:1956921. doi: 10.3389/fmed.2026.1956921 (PMC13690682; doi:10.3389/fmed.2026.1956921)
Supplement: Supplementary file 1 [file Table1.docx]

Supplementary Table 1. Candidate predictors and variable definitions

| **Predictor group** | **Standardized variable** | **Display name** | **Full term / definition** | **Unit / coding** | **Primary model** | **Included in any reported sensitivity analysis** | **Missing indicator in primary model** | **Model inclusion status / notes** | **Cleaning / additional notes** |
| --- | --- | --- | --- | --- | --- | --- | --- | --- | --- |
| Primary predictor | age | Age | Age in years | years | Yes | Yes | age_missing | Included in the primary model | Values outside 0–120 set to missing |
| Primary predictor | sex | Sex | Sex coded as male/female | male/female | Yes | Yes | sex_missing | Included in the primary model | Unrecognized values set to missing |
| Primary predictor | BMI | BMI | Body mass index | kg/m² | Yes | Yes | BMI_missing | Included in the primary model | Values outside 5–80 set to missing |
| Primary predictor | PCT | PCT | Procalcitonin | ng/mL | Yes | Yes | PCT_missing | Included in the primary model | Values outside 0–1000 set to missing |
| Primary predictor | WBC | WBC | White blood cell count | ×10⁹/L | Yes | Yes | WBC_missing | Included in the primary model | Values outside 0–200 set to missing |
| Primary predictor | PLT | PLT | Platelet count | ×10⁹/L | Yes | Yes | PLT_missing | Included in the primary model | Values outside 0–2000 set to missing |
| Primary predictor | NEU_R | Neutrophil ratio | Neutrophil ratio, standardized as percentage | % | Yes | Yes | NEU_R_missing | Included in the primary model | Percentage or percentage-like scale retained; 0–100 plausibility range |
| Primary predictor | hsCRP | hsCRP | High-sensitivity C-reactive protein | mg/L | Yes | Yes | hsCRP_missing | Included in the primary model | Values outside 0–500 set to missing |
| Primary predictor | Na | Sodium | Serum sodium | mmol/L | Yes | Yes | Na_missing | Included in the primary model | Values outside 80–180 set to missing |
| Primary predictor | K | Potassium | Serum potassium | mmol/L | Yes | Yes | K_missing | Included in the primary model | Values outside 1–10 set to missing |
| Primary predictor | ALT | ALT | Alanine aminotransferase | U/L | Yes | Yes | ALT_missing | Included in the primary model | Values outside 0–10000 set to missing |
| Primary predictor | TBIL | TBIL | Total bilirubin | µmol/L | Yes | Yes | TBIL_missing | Included in the primary model | Values outside 0–1000 set to missing |
| Primary predictor | ALB | Albumin | Albumin | g/L | Yes | Yes | ALB_missing | Included in the primary model | Values outside 0–100 set to missing |
| Primary predictor | PT | PT | Prothrombin time | s | Yes | Yes | PT_missing | Included in the primary model | Values outside 5–200 set to missing |
| Initially considered predictor | ESR | ESR | Erythrocyte sedimentation rate | mm/h | No | No | No | Excluded from all final reported models because of high missingness | Overall missingness 81.07% |
| Initially considered predictor | LA | Lactate | Lactate | mmol/L | No | No | No | Excluded from all final reported models because of very high missingness | Overall missingness 93.48% |
| Excluded predictor | HbA1c | HbA1c | Glycated hemoglobin | % | No | No | No | Excluded because of extreme or nearly complete missingness | Overall missingness 99.65% |
| Excluded predictor | AG | Anion gap | Anion gap | mmol/L | No | No | No | Excluded because of extreme or nearly complete missingness | Overall missingness 99.65% |
| Excluded predictor | Crea | Creatinine | Creatinine | µmol/L | No | No | No | Excluded because of extreme or nearly complete missingness | Overall missingness 99.38% |
| Excluded predictor | D_Di | D-dimer | D-dimer | as recorded in LIS | No | No | No | Excluded because of extreme or nearly complete missingness | Overall missingness 100.00% |

The primary model included 14 original clinical predictors and their corresponding missingness indicators.

Age, PCT, and BMI were additionally evaluated together in an exploratory post hoc logistic comparator after SHAP analysis identified them as the three dominant contributors in the 2024 validation cohort. This three-predictor set was not part of the original Phase 4/5 candidate-model pipeline and its temporal-validation performance should not be interpreted as independent validation of a prespecified predictor set.
