# Supplementary material for: Development and temporal validation of a calibrated machine learning model for in-hospital blood culture positivity using routine clinical data
Source: Front Med (Lausanne). 2026 Oct 9;13:1956921. doi: 10.3389/fmed.2026.1956921 (PMC13690682; doi:10.3389/fmed.2026.1956921)
Supplement: Supplementary file 2 [file Table2.docx]

Supplementary Table 2. Data-cleaning rules and implausible values

| **Variable** | **Display name** | **Unit / coding** | **Prespecified plausible range** | **Nonmissing before cleaning, n** | **Below lower bound, n** | **Above upper bound, n** | **Set to missing, n** | **Set to missing among nonmissing, %** | **Cleaning action / rule note** |
| --- | --- | --- | --- | --- | --- | --- | --- | --- | --- |
| age | Age | years | 0–120 | 43,881 | 41 | 0 | 41 | 0.093% | Age outside 0–120 years was considered implausible. |
| sex | Sex | male/female | Recognized male/female coding only | NA | NA | NA | NA | NA | Unrecognized sex values were set to missing and represented by sex_missing in the primary model. |
| BMI | BMI | kg/m² | 5–80 | 30,317 | 0 | 0 | 0 | 0.000% | BMI outside 5–80 kg/m² was considered implausible. |
| PCT | PCT | ng/mL | 0–1000 | 41,987 | 0 | 0 | 0 | 0.000% | PCT outside 0–1000 was considered implausible. |
| WBC | WBC | ×10⁹/L | 0–200 | 26,372 | 0 | 3 | 3 | 0.011% | WBC outside 0–200 was considered implausible. |
| PLT | PLT | ×10⁹/L | 0–2000 | 26,373 | 0 | 0 | 0 | 0.000% | PLT outside 0–2000 was considered implausible. |
| NEU_R | Neutrophil ratio | % | 0–100 | 26,329 | 0 | 0 | 0 | 0.000% | NEU_R outside 0–100% was considered implausible. |
| hsCRP | hsCRP | mg/L | 0–500 | 26,355 | 0 | 0 | 0 | 0.000% | hsCRP outside 0–500 was considered implausible. |
| Na | Sodium | mmol/L | 80–180 | 38,128 | 1 | 2 | 3 | 0.008% | Sodium outside 80–180 mmol/L was considered implausible. |
| K | Potassium | mmol/L | 1–10 | 38,099 | 0 | 30 | 30 | 0.079% | Potassium outside 1–10 mmol/L was considered implausible. |
| ALT | ALT | U/L | 0–10000 | 19,308 | 0 | 0 | 0 | 0.000% | ALT outside 0–10000 was considered implausible. |
| TBIL | TBIL | µmol/L | 0–1000 | 19,305 | 2 | 0 | 2 | 0.010% | TBIL outside 0–1000 was considered implausible. |
| ALB | Albumin | g/L | 0–100 | 19,311 | 0 | 0 | 0 | 0.000% | ALB outside 0–100 was considered implausible. |
| PT | PT | s | 5–200 | 18,858 | 0 | 0 | 0 | 0.000% | PT outside 5–200 seconds was considered implausible. |
| ESR | ESR | mm/h | 0–200 | 8,404 | 0 | 0 | 0 | 0.000% | ESR outside 0–200 was considered implausible. |
| LA | Lactate | mmol/L | 0–30 | 2,900 | 0 | 7 | 7 | 0.241% | Lactate outside 0–30 mmol/L was considered implausible for primary cleaning. |
| HbA1c | HbA1c | % | Not applied for final model development | NA | NA | NA | NA | NA | Excluded before model development because of extreme or almost complete missingness. |
| AG | Anion gap | mmol/L | Not applied for final model development | NA | NA | NA | NA | NA | Excluded before model development because of extreme or almost complete missingness. |
| Crea | Creatinine | µmol/L | Not applied for final model development | NA | NA | NA | NA | NA | Excluded before model development because of extreme or almost complete missingness. |
| D_Di | D-dimer | as recorded in LIS | Not applied for final model development | NA | NA | NA | NA | NA | Excluded before model development because of extreme or almost complete missingness. |

For age, 41 values outside the prespecified 0–120-year range were set to missing in the full analytic cohort. All 41 occurred in the 2022–2023 development cohort; none occurred in the 2024 temporal validation cohort.

The 150 age-missing records in the 2024 temporal validation cohort were originally missing/blank rather than created by the plausibility-range cleaning rule.
