# Supplementary material for: Development and temporal validation of a calibrated machine learning model for in-hospital blood culture positivity using routine clinical data
Source: Front Med (Lausanne). 2026 Oct 9;13:1956921. doi: 10.3389/fmed.2026.1956921 (PMC13690682; doi:10.3389/fmed.2026.1956921)
Supplement: Supplementary file 3 [file Table3.docx]

**Supplementary Table 3. Missingness of candidate predictors**

| **Predictor group** | **Variable** | **Display name** | **Overall analytic cohort missing, n (%)** | **Development cohort missing, n (%)** | **Temporal validation cohort missing, n (%)** | **New-patient temporal validation subset missing, n (%)** |
| --- | --- | --- | --- | --- | --- | --- |
| Primary predictor | age | Age | 549 (1.24%) | 399 (1.23%) | 150 (1.26%) | 139 (1.29%) |
| Primary predictor | sex | Sex | 508 (1.14%) | 358 (1.10%) | 150 (1.26%) | 139 (1.29%) |
| Primary predictor | BMI | BMI | 14,072 (31.70%) | 10,775 (33.16%) | 3,297 (27.71%) | 3,164 (29.34%) |
| Primary predictor | PCT | PCT | 2,402 (5.41%) | 1,741 (5.36%) | 661 (5.56%) | 617 (5.72%) |
| Primary predictor | WBC | WBC | 18,020 (40.60%) | 13,080 (40.26%) | 4,940 (41.52%) | 4,452 (41.28%) |
| Primary predictor | PLT | PLT | 18,016 (40.59%) | 13,079 (40.25%) | 4,937 (41.49%) | 4,450 (41.26%) |
| Primary predictor | NEU_R | Neutrophil ratio | 18,060 (40.69%) | 13,105 (40.33%) | 4,955 (41.65%) | 4,462 (41.37%) |
| Primary predictor | hsCRP | hsCRP | 18,034 (40.63%) | 13,093 (40.30%) | 4,941 (41.53%) | 4,453 (41.29%) |
| Primary predictor | Na | Sodium | 6,264 (14.11%) | 4,541 (13.98%) | 1,723 (14.48%) | 1,656 (15.35%) |
| Primary predictor | K | Potassium | 6,320 (14.24%) | 4,579 (14.09%) | 1,741 (14.63%) | 1,673 (15.51%) |
| Primary predictor | ALT | ALT | 25,081 (56.50%) | 17,457 (53.73%) | 7,624 (64.08%) | 6,789 (62.95%) |
| Primary predictor | TBIL | TBIL | 25,086 (56.51%) | 17,461 (53.74%) | 7,625 (64.09%) | 6,789 (62.95%) |
| Primary predictor | ALB | Albumin | 25,078 (56.50%) | 17,455 (53.72%) | 7,623 (64.07%) | 6,788 (62.94%) |
| Primary predictor | PT | PT | 25,531 (57.52%) | 18,622 (57.31%) | 6,909 (58.07%) | 6,135 (56.88%) |
| Initially considered predictor | ESR | ESR | 35,985 (81.07%) | 26,705 (82.19%) | 9,280 (78.00%) | 8,402 (77.90%) |
| Initially considered predictor | LA | Lactate | 41,496 (93.48%) | 30,303 (93.27%) | 11,193 (94.07%) | 10,142 (94.04%) |
| Excluded predictor | HbA1c | HbA1c | 44,234 (99.65%) | 32,465 (99.92%) | 11,769 (98.92%) | 10,664 (98.88%) |
| Excluded predictor | AG | Anion gap | 44,234 (99.65%) | 32,465 (99.92%) | 11,769 (98.92%) | 10,664 (98.88%) |
| Excluded predictor | Crea | Creatinine | 44,114 (99.38%) | 32,273 (99.33%) | 11,841 (99.52%) | 10,729 (99.48%) |
| Excluded predictor | D_Di | D-dimer | 44,389 (100.00%) | 32,491 (100.00%) | 11,898 (100.00%) | 10,785 (100.00%) |

Missingness was calculated after data cleaning and before imputation.

In the 2024 temporal validation cohort, age was missing in 150 records (1.26%); 24 were outcome-positive (event rate, 16.0%). All 150 age-missing records were originally missing/blank; none resulted from the 0–120-year plausibility-range cleaning rule. All 41 out-of-range age values in the full analytic cohort occurred in the development cohort.

Sex was also missing in 150 validation records (1.26%); 24 were outcome-positive (16.0%). All 150 records with missing sex also had missing age, representing complete overlap; there were no records with isolated missing sex or isolated missing age.

ESR and lactate were excluded from all final reported models because of high or very high missingness. HbA1c, anion gap, creatinine, and D-dimer were excluded because of extreme or nearly complete missingness.
