# Supplementary material for: Development and temporal validation of a calibrated machine learning model for in-hospital blood culture positivity using routine clinical data
Source: Front Med (Lausanne). 2026 Oct 9;13:1956921. doi: 10.3389/fmed.2026.1956921 (PMC13690682; doi:10.3389/fmed.2026.1956921)
Supplement: Supplementary file 4 [file Table4.docx]

Supplementary Table 4. XGBoost hyperparameter tuning results in the development cohort

| **Selected final model** | **Grid ID** | **max_depth** | **eta** | **min_child_weight** | **subsample** | **colsample_bytree** | **gamma** | **lambda** | **alpha** | **scale_pos_weight** | **Best iteration** | **Mean CV PR-AUC** | **SD CV PR-AUC** |
| --- | --- | --- | --- | --- | --- | --- | --- | --- | --- | --- | --- | --- | --- |
| Yes | 6 | 3 | 0.03 | 5 | 0.80 | 0.80 | 0.00 | 1.00 | 0.00 | 11.210 | 155 | 0.276 | 0.008 |
| No | 8 | 3 | 0.05 | 5 | 0.80 | 0.80 | 0.00 | 1.00 | 0.00 | 11.210 | 104 | 0.276 | 0.010 |
| No | 2 | 3 | 0.03 | 1 | 0.80 | 0.80 | 0.00 | 1.00 | 0.00 | 11.210 | 151 | 0.275 | 0.009 |
| No | 4 | 3 | 0.05 | 1 | 0.80 | 0.80 | 0.00 | 1.00 | 0.00 | 11.210 | 106 | 0.274 | 0.008 |
| No | 7 | 2 | 0.05 | 5 | 0.80 | 0.80 | 0.00 | 1.00 | 0.00 | 11.210 | 160 | 0.270 | 0.010 |
| No | 1 | 2 | 0.03 | 1 | 0.80 | 0.80 | 0.00 | 1.00 | 0.00 | 11.210 | 200 | 0.269 | 0.012 |
| No | 5 | 2 | 0.03 | 5 | 0.80 | 0.80 | 0.00 | 1.00 | 0.00 | 11.210 | 193 | 0.269 | 0.011 |
| No | 3 | 2 | 0.05 | 1 | 0.80 | 0.80 | 0.00 | 1.00 | 0.00 | 11.210 | 73 | 0.265 | 0.009 |
| Tuning was performed in the development cohort using patient-grouped five-fold cross-validation. | | | | | | | | | | | | | |
| The primary optimization metric was PR-AUC. | | | | | | | | | | | | | |
| Class imbalance was addressed using scale_pos_weight. | | | | | | | | | | | | | |
