# Supplementary material for: Development and temporal validation of a calibrated machine learning model for in-hospital blood culture positivity using routine clinical data
Source: Front Med (Lausanne). 2026 Oct 9;13:1956921. doi: 10.3389/fmed.2026.1956921 (PMC13690682; doi:10.3389/fmed.2026.1956921)
Supplement: Supplementary file 5 [file Table5.docx]

Supplementary Table 5. Additional calibration analyses of the XGBoost model

| **Calibration** | **Development ROC-AUC (95% CI)** | **Development PR-AUC** | **Development Brier score** | **Validation ROC-AUC (95% CI)** | **Validation PR-AUC** | **Validation Brier score** | **Validation log loss** | **Mean predicted risk** | **O/E ratio** | **Calibration intercept / slope** |
| --- | --- | --- | --- | --- | --- | --- | --- | --- | --- | --- |
| Uncalibrated | 0.782 (0.773–0.791) | 0.271 | 0.1856 | 0.798 (0.784–0.813) | 0.266 | 0.1699 | 0.5120 | 37.30% | 0.20 | -2.377 / 1.136 |
| Platt | 0.782 (0.773–0.791) | 0.271 | 0.0672 | 0.798 (0.784–0.813) | 0.266 | 0.0619 | 0.2230 | 7.44% | 1.01 | 0.137 / 1.061 |
| Isotonic | 0.784 (0.776–0.793) | 0.278 | 0.0669 | 0.797 (0.782–0.811) | 0.262 | 0.0620 | 0.2233 | 7.41% | 1.01 | 0.112 / 1.047 |
| Development-cohort values represent patient-grouped five-fold out-of-fold predictions; validation values refer to the independent 2024 temporal validation cohort. | | | | | | | | | | |
| The uncalibrated XGBoost outputs were influenced by class weighting and were not interpreted as directly usable clinical probabilities. | | | | | | | | | | |
| Platt calibration was prespecified as the primary calibration approach. Isotonic calibration was evaluated as a sensitivity analysis. | | | | | | | | | | |
| Abbreviations: ROC-AUC, area under the receiver operating characteristic curve; PR-AUC, area under the precision-recall curve; O/E, observed-to-expected; CI, confidence interval. | | | | | | | | | | |
