# Supplementary material for: Development and temporal validation of a calibrated machine learning model for in-hospital blood culture positivity using routine clinical data
Source: Front Med (Lausanne). 2026 Oct 9;13:1956921. doi: 10.3389/fmed.2026.1956921 (PMC13690682; doi:10.3389/fmed.2026.1956921)
Supplement: Supplementary file 6 [file Table6.docx]

**Supplementary Table 6. Additional age-related exploratory and sensitivity analyses in the 2024 temporal validation cohort**

| **Analysis** | **Validation set / subgroup** | **Model** | **Events / N** | **Event rate** | **Mean predicted risk** | **O/E ratio** | **ROC-AUC (95% CI)** | **PR-AUC** | **Brier score** | **Calibration slope** |
| --- | --- | --- | --- | --- | --- | --- | --- | --- | --- | --- |
| Predictor-removal sensitivity | Overall 2024 temporal validation cohort | XGBoost without age | 894 / 11,898 | 7.51% | 7.74% | 0.97 | 0.788 (0.773–0.803) | 0.261 | 0.0623 | 1.076 |
| No-age subgroup evaluation | Adult subgroup, age ≥18 years | XGBoost without age | 739 / 5,172 | 14.29% | 13.02% | 1.10 | 0.673 (0.651–0.695) | 0.293 | 0.1143 | 0.894 |
| No-age subgroup evaluation | Pediatric subgroup, age <18 years | XGBoost without age | 131 / 6,576 | 1.99% | 3.42% | 0.58 | 0.657 (0.606–0.709) | 0.068 | 0.0197 | 0.813 |
| No-age subgroup evaluation | Age-missing subgroup | XGBoost without age | 24 / 150 | 16.00% | 15.18% | 1.05 | 0.500 (0.475–0.525) | 0.151 | 0.1372 | -0.870 |
| Predictor-removal sensitivity | Overall 2024 temporal validation cohort | XGBoost without age and all missingness indicators | 894 / 11,898 | 7.51% | 7.83% | 0.96 | 0.785 (0.770–0.799) | 0.255 | 0.0625 | 1.074 |
| Predictor-removal sensitivity | Overall 2024 temporal validation cohort | XGBoost without age and BMI | 894 / 11,898 | 7.51% | 7.72% | 0.97 | 0.773 (0.757–0.789) | 0.254 | 0.0627 | 1.060 |
| Age-specific exploratory model | Adult subgroup, age ≥18 years | Adult-only XGBoost | 739 / 5,172 | 14.29% | 13.55% | 1.05 | 0.679 (0.658–0.701) | 0.302 | 0.1137 | 0.972 |
| Age-specific exploratory model | Pediatric subgroup, age <18 years | Pediatric-only XGBoost | 131 / 6,576 | 1.99% | 2.42% | 0.82 | 0.649 (0.602–0.695) | 0.058 | 0.0192 | 0.967 |
| Age-standardized discrimination | Known-age validation records | Equal-weight adult/pediatric standardized AUC | 870 / 11,748 |  |  |  | 0.671 (0.645–0.696) |  |  |  |

For XGBoost without age, both age and its corresponding missingness indicator (age_missing) were removed, consistent with the original Phase 8 sensitivity-model definition.

The adult and pediatric no-age rows evaluate the same no-age model, trained in the overall development cohort, within the corresponding 2024 validation subgroups; these rows are distinct from the separately retrained adult-only and pediatric-only models.

For the XGBoost model without age and all missingness indicators, age and all 14 missingness indicators were removed.

For the XGBoost model without age and BMI, age, BMI, age_missing, and BMI_missing were removed.

The equal-weight adult/pediatric standardized AUC averages the adult and pediatric within-group AUCs with equal weights and excludes between-age-group comparisons.
