# Supplementary material for: Development and temporal validation of a calibrated machine learning model for in-hospital blood culture positivity using routine clinical data
Source: Front Med (Lausanne). 2026 Oct 9;13:1956921. doi: 10.3389/fmed.2026.1956921 (PMC13690682; doi:10.3389/fmed.2026.1956921)
Supplement: Supplementary file 7 [file Table7.docx]

Supplementary Table 7. Admission-ward composition of the development and temporal validation cohorts

| **Admission ward category** | **Development cohort, n (%)** | **2024 temporal validation cohort, n (%)** |
| --- | --- | --- |
| General pediatrics | 11,927 (36.71%) | 4,833 (40.62%) |
| Neonatology | 4,431 (13.64%) | 1,498 (12.59%) |
| Other medical/specialty wards | 6,369 (19.60%) | 2,324 (19.53%) |
| Surgical wards | 5,040 (15.51%) | 1,715 (14.41%) |
| Pulmonary and Critical Care Medicine | 1,734 (5.34%) | 503 (4.23%) |
| Hematology | 1,256 (3.87%) | 410 (3.45%) |
| Emergency medicine | 509 (1.57%) | 163 (1.37%) |
| Oncology | 447 (1.38%) | 183 (1.54%) |
| Obstetrics/Gynecology | 416 (1.28%) | 117 (0.98%) |
| ICU/PICU | 4 (0.01%) | 2 (0.02%) |
| Missing | 358 (1.10%) | 150 (1.26%) |
| Admission ward was determined from the electronic medical record field representing ward at hospital admission. | | |
| Admission ward does not necessarily represent the patient's ward or ICU status at the time of index blood-culture collection. | | |
| ICU/PICU therefore refers only to records whose admission ward was explicitly recorded as an ICU or PICU. | | |
| Pulmonary and Critical Care Medicine was retained as a separate admission-ward category and was not classified as ICU/PICU. | | |
| Other medical/specialty wards included remaining nonsurgical medical and specialty wards not represented by the separately reported categories. | | |
| Abbreviations: ICU, intensive care unit; PICU, pediatric intensive care unit. | | |
