# Supplementary material for: Development and temporal validation of a calibrated machine learning model for in-hospital blood culture positivity using routine clinical data
Source: Front Med (Lausanne). 2026 Oct 9;13:1956921. doi: 10.3389/fmed.2026.1956921 (PMC13690682; doi:10.3389/fmed.2026.1956921)
Supplement: Supplementary file 8 [file Table8.docx]

Supplementary Table 8. Development and calibration parameters of the exploratory Age + PCT + BMI logistic model

| **Parameter** | **Estimate** | **Exponentiated estimate (OR)** | **Estimation dataset / method** |
| --- | --- | --- | --- |
| Logistic coefficient: (Intercept) | -3.943174 |  | Full 2022–2023 development cohort after development-derived imputation |
| Logistic coefficient: age | 0.020961 | 1.021182 | Full 2022–2023 development cohort after development-derived imputation |
| Logistic coefficient: PCT | 0.024638 | 1.024944 | Full 2022–2023 development cohort after development-derived imputation |
| Logistic coefficient: BMI | 0.028387 | 1.028794 | Full 2022–2023 development cohort after development-derived imputation |
| Platt intercept | -0.005250 |  | Development OOF predictions using original Phase 4 folds |
| Platt slope | 0.997659 |  | Development OOF predictions using original Phase 4 folds |
| Temporal-validation ROC-AUC | 0.757 (0.742–0.773) |  | Independent 2024 temporal validation cohort |

The Age + PCT + BMI model was an exploratory post hoc comparator motivated by the three dominant predictors identified by SHAP analysis in the 2024 validation cohort; the predictor set was therefore not independently prespecified before viewing the temporal-validation SHAP results.

Missing age, PCT, and BMI values were imputed using parameters estimated from the 2022–2023 development cohort only.

The logistic model was fitted using development data only. Platt calibration was then fitted to patient-grouped development out-of-fold predictions using the original Phase 4 fold assignments and was fixed before application to the 2024 cohort.

The 2024 temporal validation cohort was not used to estimate imputation parameters, logistic coefficients, or Platt calibration parameters.
